# Genome-Wide Identification of Direct Targets of ZjVND7 Reveals the Putative Roles of Whole-Genome Duplication in Sour Jujube in Regulating Xylem Vessel Differentiation and Drought Tolerance

**DOI:** 10.3389/fpls.2022.829765

**Published:** 2022-02-04

**Authors:** Meng Li, Lu Hou, Chenxing Zhang, Weicong Yang, Xinru Liu, Hanqing Zhao, Xiaoming Pang, Yingyue Li

**Affiliations:** ^1^National Engineering Laboratory for Tree Breeding, College of Biological Sciences and Technology, Beijing Forestry University, Beijing, China; ^2^Key Laboratory of Genetics and Breeding in Forest Trees and Ornamental Plants, Ministry of Education, College of Biological Sciences and Technology, Beijing Forestry University, Beijing, China; ^3^The Tree and Ornamental Plant Breeding and Biotechnology Laboratory of National Forestry and Grassland Administration, Beijing Forestry University, Beijing, China

**Keywords:** autotetraploid, drought tolerance, xylem differentiation, DNA affinity purification sequencing, VND7, sour jujube

## Abstract

The effects of whole-genome duplication span multiple levels. Previous study reported that the autotetraploid sour jujube exhibited superior drought tolerance than diploid. However, the difference in water transport system between diploids and autotetraploids and its mechanism remain unclear. Here, we found the number of xylem vessels and parenchyma cells in autotetraploid sour jujube increased to nearly twice that of diploid sour jujube, which may be closely related to the differences in xylem vessel differentiation-related ZjVND7 targets between the two ploidy types. Although the five enriched binding motifs are different, the most reliable motif in both diploid and autotetraploid sour jujube was CTTNAAG. Additionally, ZjVND7 targeted 236 and 321 genes in diploids and autotetraploids, respectively. More identified targeted genes of ZjVND7 were annotated to xylem development, secondary wall synthesis, cell death, cell division, and DNA endoreplication in autotetraploids than in diploids. SMR1 plays distinct roles in both proliferating and differentiated cells. Under drought stress, the binding signal of ZjVND7 to *ZjSMR1* was stronger in autotetraploids than in diploids, and the fold-changes in the expression of *ZjVND7* and *ZjSMR1* were larger in the autotetraploids than in the diploids. These results suggested that the targeted regulation of ZjVND7 on *ZjSMR1* may play valuable roles in autotetraploids in the response to drought stress. We hypothesized that the binding of ZjVND7 to *ZjSMR1* might play a role in cell division and transdifferentiation from parenchyma cells to vessels in the xylem. This regulation could prolong the cell cycle and regulate endoreplication in response to drought stress and abscisic acid, which may be stronger in polyploids.

## Introduction

As important drivers of evolution, almost all eukaryotic genome sequences bear evidence of ancient whole-genome duplications events (Adams and Wendel, 2005). After whole-genome doubling, gene dose effects, modified regulatory interactions, and rapid genetic and epigenetic modifications and changes strongly affect the genome (Jackson and Chen, 2010). These changes affect gene expression, resulting in differences in traits such as those related to morphology, physiology, and adaptability (Allario et al., 2013; Zhang et al., 2015). Moreover, in the duplicated genome, paralogous copies may acquire a new function (neofunctionalization), several functions controlled by ancestral genes may be partitioned between duplicated genes (subfunctionalization), and redundant copies may accumulate mutations and lose their function, becoming pseudogenes (non-functional). These neofunctionalization, subfunctionalization, and pseudogenization processes are routes to adaptive diversification in polyploidy. In addition, gene regulatory networks involved in transcription factor (TF) pathways can be reconstructed by subfunctionalization and gene silencing (Osborn et al., 2003; De Smet and Van de Peer, 2012). Target genes bound by TFs are affected by the characteristics of the transcription factor binding site (TFBS) in the downstream promoter sequence. During polyploidization of a promoter region, some changes affect TF binding and thus alter the specifics of regulation. Recently, an instance of regulatory divergence among homologous genes via modulation of the TFBS profile was reported (Ye et al., 2016). Additionally, in *Brassica*, the change in the flowering time of allopolyploids is considered to be caused by deletion of the TFBS in the promoter region of the *SOC* homologous gene (Sri et al., 2020). However, compared with orthologs from different species, paralogs from the same species are better systems for studying the tempo, mode, and mechanisms of TFBS evolution. What is fascinating is how the number, function, and expression of TF target genes will be adjusted to balance this dose difference after a sudden homologous polyploidization. Therefore, a careful study of the target genes of TFs and their expression patterns will not only reveal the evolutionary mode of autopolyploid variation but also shed new light on the corresponding regulatory network.

NAC TFs, which compose one of the largest plant TF families, are involved in various processes, including plant organ development, secondary cell wall development, signal transduction, leaf senescence, and responses to biotic and abiotic stresses (Olsen et al., 2005; Puranik et al., 2012). These functions are mediated by NAC TFs in combination with multiple factors. In addition to the two important motifs of NACRS and CACG or CATGTG targeted by NACs (Xu et al., 2013; Han et al., 2021), many studies have shown that NAC TFs can also target a variety of additional motifs, including JUB1 binding site, NBE binding element (TTACGT), and ABRE motif (ACGTG), that participate in plant drought tolerance (Yuan et al., 2019; Shang et al., 2020; Li et al., 2021c). During plant growth and development, a sufficient number of vascular elements, especially the xylem, are essential to promote long-distance transport and alleviate the effects of drought. In this respect, a subfamily of NAC TFs related to the regulation of xylem differentiation called VASCULARRELATED NAC-DOMAIN (VND) has been discovered (Kubo et al., 2005). A previous study has shown that VND7 is one of the first-layer master transcriptional switches for xylem vessel differentiation (Endo et al., 2015). Its overexpression induces the ectopic differentiation of protoxylem-like vessels, while the functional suppression causes defects in the formation of vessel elements (Kubo et al., 2005; Yamaguchi et al., 2008). It has been reported that VND7 can bind to two specific regions—X1E1 and X1E2—of *XCP1* for programmed cell death (Yamaguchi et al., 2011). In addition, *PtrWND*, a homolog of *VND7*, has been shown to regulate the expression of genes related to xylem cell differentiation, programmed cell death, enzymes, and signal transduction pathways (Ohtani et al., 2011). Nonetheless, variations in and mechanisms of xylem vessel differentiation in polyploids remain poorly understood.

Chinese jujube (*Ziziphus jujuba* Mill.), which is native to China, is known for its nutritious, medicinally valuable, edible fruits (Guo and Shan, 2010; Keles, 2020). Excellent jujube varieties are generally propagated by grafting, and the drought and saline-alkali tolerance of these trees depends on the rootstock (Liu, 2010). Owing to its tolerance of poor environmental conditions, sour jujube (Z. *jujuba* var. *spinosa*) has been widely used as a rootstock tree (Wang and Sun, 1986). Many TFs in jujube, including WRKY (Chen et al., 2019), DREB (Zhou et al., 2019), and NAC (Li et al., 2021a). TFs, have been reported to be differentially expressed in response to drought stress, but their underlying is still unclear. In a previous study, autotetraploid sour jujube was acquired by colchicine-induced somatic cell chromosomal doubling, and superior salt resistance and drought tolerance were detected in autotetraploid sour jujube compared with diploid sour jujube (Li et al., 2019c, 2021b). Furthermore, the type and number of TFs changed between the diploid and autotetraploid plants, especially NAC TFs after salinity and drought stress. In the present study, differences in xylem vessels were characterized between diploid and autotetraploid sour jujube. A presumptive *ZjVND7* gene involved in vessel differentiation was differentially expressed between diploid and autotetraploid sour jujube under drought conditions. We were interested in determining how the numbers and functions of downstream target genes regulated by ZjVND7 changed after whole-genome duplication. Therefore, a genome-wide investigation of ZjVND7 target genes in the genomes of diploid and autotetraploid sour jujube under drought stress was conducted through DNA affinity purification sequencing (DAP-seq). Differences in binding motifs between diploid and autotetraploid plants were identified, and the enriched functions of target genes were further analyzed. In addition, *ZjSMR1* targeted by ZjVND7 was identified, and its putative effects on vessel differentiation regulation and the drought response were proposed. Our research provides evidence for the diversified adjustment of plant genomes in response to polyploidization. The putative functions of ZjVND7 and its target genes and the related genetic pathways may be useful tools for designing plant drought tolerance in the future.

## Materials and Methods

### Plant Materials and Treatments

Two-year-old diploid and autotetaploid sour jujube plants growing in pots and tissue cultural seedlings maintained for more than 20 generations were preserved in the National Engineering Laboratory for Tree Breeding, Beijing Forestry University, Beijing, China. Two-year-old plants were grown outdoors, and the tissue cultural materials were grown under a 16/8 h light/dark cycle at 25 ± 2°C and under 130 μmol/m^2^/s illumination. More than 50 and 30 diploid and autotetraploid tissue cultural seedlings, respectively, were cultured in rooting medium for 45 days. Then, diploids and autotetraploids displaying uniform growth were transplanted into vermiculite soil and subsequently grown in a greenhouse with a temperature of 24 ± 1°C under a 16 h photoperiod with a cool-white fluorescent light (3,000 lx). After 30 days, the diploids and autotetraploids were subjected to drought and sprayed with abscisic acid (ABA). For drought treatment, after watering through, not watered until the soil water content reaches 4% and held for 7 days. For ABA treatment, the aboveground parts of diploid sour jujube were sprayed with 10 mg/L ABA every 3 h. Mature leaves (leaf order 4–6 from the shoot apex) of diploid and autotetraploid sour jujube plants after drought treatment and after 2 and 12 h of ABA spraying were collected and snap-frozen in liquid nitrogen. For each experiment, there were four plants per replication, and each treatment was repeated three times.

### Anatomical Analyses

Fourth-order leaves of 2-year-old diploids and autotetraploids were collected and cut into paraffin sections, with five biological repeats. All the leaves were preserved in FAA solution (50% ethyl alcohol, formaldehyde and glacial acetic acid V:V:V = 18:1:1). Dehydration was performed using ethanol and xylene at different concentrations. Then, the leaves were embedded in paraffin wax. The wax was cut into 8 μm sections and then dyed with safranine and fast green dyes. After decolorization with picric acid, the leaves were observed under a Nikon Eclipse Ci microscope (Nikon, Tokyo, Japan). SPSS 20.0 statistical software (IBM, New York, NY, United States) was used to analyze the number of vessels and parenchyma cells in the xylem. Significant differences were determined via *t*-tests, with * indicating *p* < 0.05 and ^**^ indicating *p* < 0.01.

### Isolation of ZjVND7 and Phylogenetic Analysis

Total RNA was extracted with a Plant RNA Kit (Omega Biotech, New York, NY, United States) and purified with RNase-free DNase set. Next, first-strand cDNA was reverse transcripted in a reaction with 1.5 μg of total RNA. The full-length coding sequence (CDS) was obtained from the *Z. jujuba* genome and cloned via PCR of the cDNA. The primer sequences used are as follows: forward, 5′-ATGGAAATGGAATCTTGTGTCCCA-3′, and reverse, 5′-CTACAAATCAGGAAAACAACCAAGA-3′. The PCR products were extracted from the gels using a Gel Extraction Kit (Tsingke, Beijing, China), and then ligated into a pMD19-T vector (Aidlab, Beijing, China) for sequencing. The orthologous genes sequences of *ZjVND7* were obtained by BLAST searches of The Arabidopsis Information Resource (TAIR) website^1^. Multiple sequence alignments were performed using ClustalW, and subsequent phylogenetic analyses were conducted with MEGAX using the maximum likelihood method (Kumar et al., 2018).

### Gene Expression Levels According to qRT-PCR

To analyze gene expression levels, qRT-PCR was carried out with a 2 × SYBR Green qPCR Mix Kit (Aidlab, China) in a 25 μl volume on a 7500 Fast Real-Time instrument (Thermo Fisher, Waltham, MA, United States) using the following cycling protocol: 3 min at 94°C followed by 40 cycles of 20 s at 94°C, 20 s at 55°C, and 30 s at 72°C (signal acquisition at 72°C). *ZjActin* was used as a reference gene (Bu et al., 2016). The 2^–ΔΔCt^ method was used to calculate the relative gene expression levels (Livak and Schmittgen, 2001).

### DAP-seq, Data Analysis and Functional Annotation

DAP-seq in diploid and autotetraploid sour jujube were performed according to the procedure published by Bartlett et al. (2017). For the DNA DAP library, fresh leaves from diploid and autotetraploid plants after drought stress were sampled, and genomic DNA was obtained using a DNA Extract Kit (Tiangen, Beijing, China) according to the manufacturer’s instructions. Then, genomic DNA was fragmented to a size of 200 bp, after which end repair, A-tailing reaction, and adapter ligation were performed. To induce the binding of proteins to magnetic beads, the full-length CDS of *ZjVND7* was first reassembled into a pFN19K HaloTag T7 SP6 Flexi vector. TNT SP6 High-Yield Protein Expression System (Promega, Wisconsin, United States) was performed to express the Halo-ZjVND7 fusion protein according to the manufacturer’s specifications (Promega, United States). Then, the expressed proteins were removed with Magne Halo Tag Beads (Promega, Madison, WI, United States). The protein-bound beads were then washed three times with buffer. For recognition of DNA bound to proteins, the beads were resuspended, and a DNA library was added to these reactions. The bound protein-bead-DNA complex was washed and then heated to 98°C for 10 min to denature the Halo-ZjVND7 proteins and release the bound DNA. Finally, a 50 μl PCR mixture that included 25 μl of released DNA was implemented.

An ∼200–400 bp DNA gel was cut out and extracted. The DNA was sequenced using an Illumina NavoSeq instrument with 100-bp single-end reads. For read alignment, the sequences in FASTQ files were aligned to the *Z. jujuba* ‘Dongzao’ genome using Bowtie 2 (Langmead and Salzberg, 2012). Read trimming and quality/repeat read filtering were further performed. For peak analysis, mapped read files (SAM or BAM format) were used to identify peaks using peak calling via MACS2 (Zhang et al., 2008). Based on general feature format (GFF) files, the peak at 3.5 kb relative to the transcription start site (TSS) was located using Homer (Heinz et al., 2010). Genes located 2.0 kb upstream were described as target genes. These genes were annotated via the Gene Ontology (GO) database (Ashburner et al., 2000), and Kyoto Encyclopedia of Genes and Genomes (KEGG) pathway enrichment analysis was performed using KOBAS 2.0 (Xie et al., 2011). Negative control mock DAP-seq libraries (inputs) were prepared without the addition of proteins to the beads. Peaks relative to those of the negative control sample were compared, and motifs were identified using MEME-ChIP 5.0.5 (Machanick and Bailey, 2011).

### Differential Expression Analysis of Diploid and Autotetraploid Sour Jujube Based on Published RNA-seq Data

In a previous study, we obtained transcriptomes from diploid and autotetraploid leaves (Li et al., 2019b). On this basis, we reanalyzed the genes that were the differential expression of genes involved in xylem differentiation, secondary cell wall development, cell cycle cell division, and cell death and that were differentially expressed between diploid and autotetraploid leaves. These differentially expressed genes were subsequently visualized in heat maps using TBtools software (Chen et al., 2020).

### Subcellular Localization

*ZjVND7* and *ZjSMR1* were recombined and ligated into a pYBA1152 vector labeled with eGFP using a one-step seamless cloning kit (Aidlab, China). In short, primers with overlapping sequences were designed for PCR amplification of full-length CDS sequences. Then, the gel was recycled, and a reaction (10 μl volume) was performed to ultimately connect it to the carrier according to the instructions. The correctly sequenced plasmid was transformed into *Agrobacterium*. Then, an MES suspension (OD = 0.5–1.0) was injected into 1-month-old tobacco leaves. The plants were incubated in the dark for 24 h under a 16/8 h light/dark cycle for 48h at 25 ± 2°C. The injection sites were then removed and observed under a SP8 microscope (Leica, Wetzlar, Germany).

### Yeast One-Hybrid Assays

The CDS of *ZjVND7* and the 474 bp DNA fragment of the *ZjSMR1* promoter region were inserted into pB42AD and pLacZi2μ vectors (Takara, Kyoto, Japan), respectively, by seamless cloning. The primer sequences used for the 474 bp DNA fragment of the *SMR1* promoter are as follows: forward, 5′-GTAGGGAAAAATGAAAACAAAAACA-3′, and reverse, 5′-TGACAGAGTAAATTGCCATTACCGT-3′. The plasmids were transformed into yeast strain EGY48. The following four combinations were used for cotransformation: pB42AD and pLacZi2μ empty vectors; a pB42AD empty vector and p*ZjSMR1*^pro(474 bp)^:lacZ; pB42AD:*ZjVND7* and a pLacZi2μ empty vector; and pB42AD:*ZjVND7* and p*ZjSMR1*^pro(474 bp)^:*lacZ*. The transformed strains were suspended in double distilled water (ddH_2_O) and then coated onto tryptophan (Trp) and uracil (Ura)-deficient synthetic dextrose (SD) medium (SD/–Trp/–Ura) for culture at 29°C. The chromogenic substrate 5-bromo-4-chloro-3-indolyl- beta-D-galactopyranoside (X-Gal) was used to characterize β-galactosidase (lacZ) reporter gene expression in the pLacZi2μ vector. The cotransformed strain was dipped and suspended in 200 μl ddH_2_O and then spotted onto SD/-Trp/-Ura medium including 80 mg/L X-Gal at 29°C. The plaque was imaged as it turned blue.

## Results

### Xylem Vessel Differences in Diploid and Autotetraploid Sour Jujube and Identification of *ZjVND7*

**FIGURE 1:**
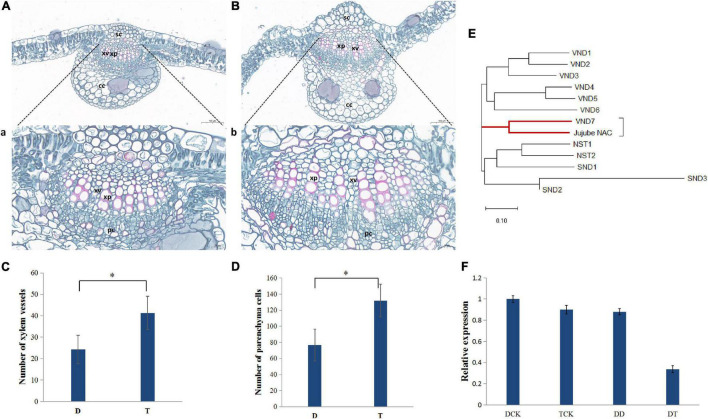
Differences in xylem between diploid and autotetraploid sour jujube and the expression levels and phylogeny of *ZjVND7*. **(A,B)** Characterization of the anatomical structure of diploids **(A)** and autotetraploids **(B)**. a, the partial view of **(A)**; b, the partial view of **(B)**. **(C,D)** Comparison of cell numbers in xylem vessels **(C)** and xylem parenchyma **(D)** in the main veins of leaves. For the difference between D and T, * represents a significance level of 0.05. **(E)** Phylogenetic analysis of jujube LOC107430472 and the *A. thaliana SWN* genes. **(F)** Expression level of *ZjVND7* in diploid and autotetraploid sour jujube under drought stress. D, diploid; T, autotetraploid; DCK, diploid before drought treatment; TCK, autotetraploid before drought treatment; DD, diploid after drought treatment; TD, autotetraploid after drought treatment; sc, sclerenchyma cell; xp, xylem parenchyma; xv, xylem vessel; cc, collenchyma cell; pc, phloem cell.

Based on the differences in morphology, the main veins of the leaves of autotetraploid sour jujube were significantly enlarged compared with those of diploid sour jujube (Figures 1A,B and Supplementary Figure 1). Additionally, the number of xylem vessels in autotetraploids increased to a number nearly twice that in diploids, while the xylem vessel size did not increase significantly in autotetraploids (Figures 1A–C and Supplementary Figure 2). Furthermore, the number of parenchyma cells markedly increased in autotetraploids (Figure 1D).

The most important physiological function of vessels is water transport. Here, when the plants were subjected to a soil water content equal to 4% field capacity for 7 days, *LOC107430472* exhibited a greater fold-changes in the autotetraploid leaves than in the diploid leaves (Figure 1F). Phylogenetic analysis confirmed that the protein encoded by *LOC107430472* was closely related to the *Arabidopsis thaliana* group secondary wall NACs (SWNs) and most strongly related to *VND7* (Figure 1E). On this basis, the *LOC107430472* was designated *ZjVND7*.

### Genome-Wide Identification of the Direct Downstream Targets of ZjVND7 in Diploid and Autotetraploid Plants

**FIGURE 2:**
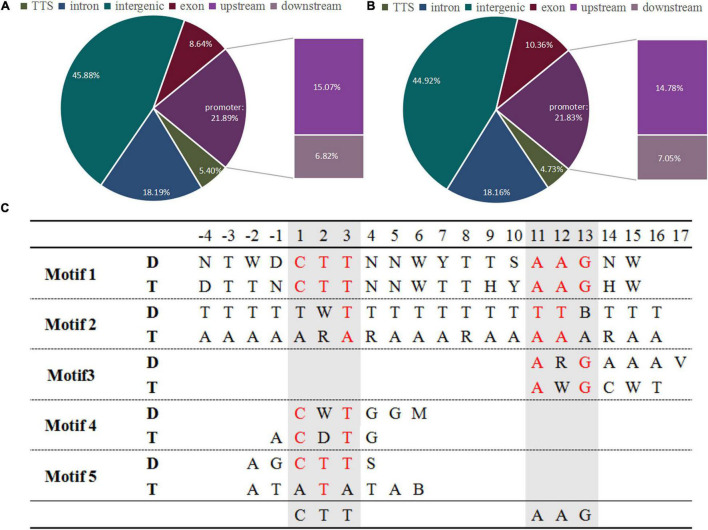
Genome-wide distribution of ZjVND7 binding peaks and types of motifs bound to the promoter in diploid and autotetraploid sour jujube. **(A,B)** Genome-wide distribution of ZjVND7 binding peaks in diploid **(A)** and autotetraploid **(B)** plants. TSS, transcription start site. **(C)** Comparison of the ZjVND7-binding motifs in diploid and autotetraploid plants. D, diploid; T, autotetraploid.

To investigate the changes in ZjVND7 interactions between diploid and autotetraploid plants under drought stress. The DAP-seq was performed on diploids and autotetraploids under drought treatment. A total of 1,759/2,538 peaks of *ZjVND7* targets identified in diploid/autotetraploid sour jujube were distributed across 12 chromosomes (Supplementary Figure 3). The number of peaks distributed throughout the gene body and its upstream and downstream in autotetraploids was significantly greater than that in diploids, but there was no significant difference in the distribution of the detected positions. As shown in Figures 2A,B, within the genic peaks, ZjVND7 showed the highest preference for binding to proximal promoter regions located up to 2.0 kb upstream of the TSS. ZjVND7 also showed preferential binding to exons and distal downstream regions that constituted 8.64 and 6.82% of all the peaks, respectively, in diploid and 10.36 and 7.05% of all the peaks, respectively, in autotetraploids. In diploids, 1,495 peaks mapped to 1,370 genes, and 265 peaks were located in promoter regions of 236 jujube genes and 26 unaligned genes (Supplementary Table 1). In the autotetraploids, 2,135 peaks corresponded to 1,915 genes, and 375 peaks were located in the promoters of 321 jujube genes and 49 unaligned genes (Supplementary Table 1). The binding motifs in target genes were divided into five types in both diploid and autotetraploid sour jujube (Figure 2C and Supplementary Table 1). The results showed that the motifs in the diploids and autotetraploids were different. However, the binding motifs in both diploid and autotetraploid sour jujube have a continuous core sequence, CTTNAAG, which is conserved with core sequence published by Tamura et al. (2019).

**FIGURE 3:**
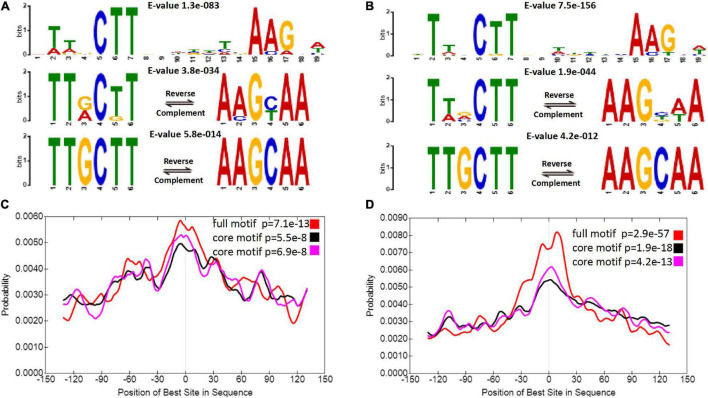
ZjVND7-bound motifs in diploid and autotetraploid sour jujube. **(A,B)** Enriched motifs within the ZjVND7 binding peaks in diploid **(A)** and autotetraploid **(B)** plants. The *E*-value was calculated by MEME. **(C,D)** Localization of the motifs relative to the ZjVND7 peak summits in diploid **(C)** and autotetraploid **(D)** plants.

In fact, the most reliable binding motif was not significantly different between diploids and autotetraploids (Figures 3A,B). Sequence analysis revealed a 19 nt motif and a TTGCTT or AAGCAA sequence, which is part of the above motif in diploids and autotetraploids. Each motif was located at a region corresponding to a peak (Figures 3C,D). Interestingly, both core motifs differed from the binding motifs previously found in *A. thaliana* VNDs (Zhong et al., 2010; O’Malley et al., 2016; Olins et al., 2018) and the 17 nt motif in maize NUT1 (Dong et al., 2020). This divergence in DNA-binding motifs suggests a putatively distinct downstream regulatory network among sour jujube, *A. thaliana* and maize.

### ZjVND7 Putative Targeted Pathways

**FIGURE 4:**
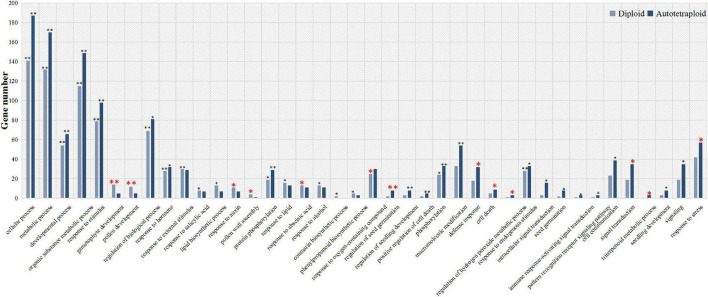
GO functional classification of ZjVND7-targeted genes. The * indicating enriched corrected *p* < 0.05 and ^**^ indicating enriched corrected *p* < 0.01.

To explore the ZjVND7-targeted functions in diploid and autotetraploid plants, GO classification and functional enrichment analysis of these target genes were performed. As shown in Figure 4, target genes in both diploid and autotetraploid plants were enriched in metabolic process, developmental process, response to stimulus, and response to hormone pathways (corrected *p*-value < 0.05). This suggests that ZjVND7 may be related to the stress response and plant development. In addition, the target genes in diploids were markedly enriched in gametophyte development, pollen development, pollen wall assembly, response to auxin, response to ABA, and response to oxygen-containing compounds. The target genes in autotetraploids were specifically enriched in the regulation of seed germination, defense response, cell death, the regulation of hydrogen peroxide metabolic process, signal transduction, and the response to stress. Moreover, the target genes in autotetraploids were annotated to the triterpenoid metabolic process, while those in diploids were not. The number of target genes annotated to the regulation of seed germination, positive regulation of cell death, regulation of hydrogen peroxide metabolic process, and immune response-activating signal transduction in autotetraploids was at least twice that in diploids. These results suggest that ZjVND7 may control a broad range of biological processes in diploid and autotetraploid sour jujube. In particular, compared with that in diploids, ZjVND7 in autotetraploids may target more pathways related to cell growth and stress signaling under drought conditions.

As shown by the KEGG annotations, the target genes in the diploid (Supplementary Table 1) and autotetraploid (Supplementary Table 1) plants were annotated in 59 and 65 pathways. A total of 27/39, 1/3, and 1/4 target genes were annotated in metabolic pathways, ABC transporters, and MAPK signaling pathway-plant pathways in diploid/autotetraploid sour jujube, respectively. The target genes in diploids were specifically annotated in 17 pathways, including carbon fixation in photosynthetic organisms and phenylalanine metabolism. In autotetraploids, targets were specifically annotated in 23 pathways, including flavonoid biosynthesis, photosynthesis, and glutathione metabolism. Taken together, these results suggest that ZjVND7 may control various stress response-related biological processes that could promote increased water transport in diploid and autotetraploid plants.

### Differential Expression of Genes Related to ZjVND7 Putative Targeted Pathways

**FIGURE 5:**
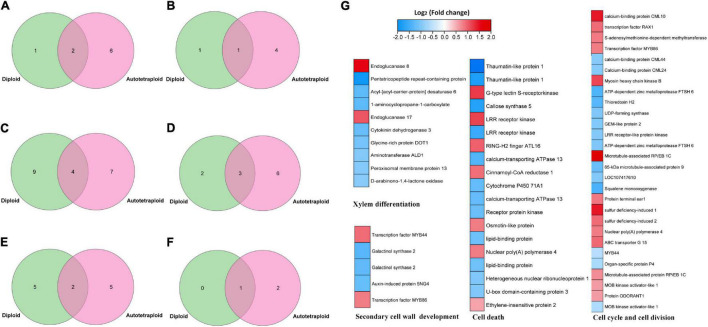
ZjVND7-targeted genes in diploid and autotetraploid sour jujube. **(A–F)** Overlap of diploid and autotetraploid ZjVND7 target genes involved in xylem development **(A)**, secondary wall synthesis **(B)**, ABA signaling **(C)**, cell death **(D)**, cell division **(E)**, and DNA replication **(F)**. **(G)** Differential expression levels of genes involved in putative ZjVND7-targeted pathways in diploids and autotetraploids under control and drought conditions.

Putative target genes of VND have been reported in *A. thaliana* (Tamura et al., 2019), maize (Dong et al., 2020), and poplar (Ohtani et al., 2011). These target genes were predicted to be related to the regulation of xylem development, programmed cell death, cell wall synthesis, metabolic enzymes, and signal transduction. Similarly, the target genes of ZjVND7 detected in this study were also predicted to participate in these pathways, but there were differences in the number of target genes between diploid and autotetraploid plants. A total of 3/8, 2/5, 13/11, 5/9, 7/7, and 1/3 target genes were detected in diploid/autotetraploid plants, respectively, and may be involved in xylem development, secondary wall synthesis, ABA signaling, cell death, cell division, and DNA replication (Figures 5A–F).

The published RNA-seq data of the leaves of diploid and autotetraploid plants under control and drought conditions were reanalyzed. Among 378 genes differentially expressed under control condition and 473 genes differentially expressed under drought conditions, 62 genes were involved in xylem differentiation, secondary cell wall development, the cell cycle and cell division, and cell death pathways, with expression fold-changes ranging from 1.6 to 6.5. In the xylem differentiation pathway, the genes encoding endoglucanase 8 and endoglucanase 17 were upregulated threefold and twofold in autotetraploids compared with diploids. In the cell cycle and cell division pathways, the gene encoding *S*-adenosylmethionine-dependent methyltransferase was upregulated twofold in the autotetraploids compared with the diploids. Taken together, these results indicated that *ZjVND7*-targeted pathways may differ in response to drought between diploid and autotetraploid sour jujube.

### ZjVND7 Directly Controls the Putative Cell Division-Related *ZjSMR1*

**FIGURE 6:**
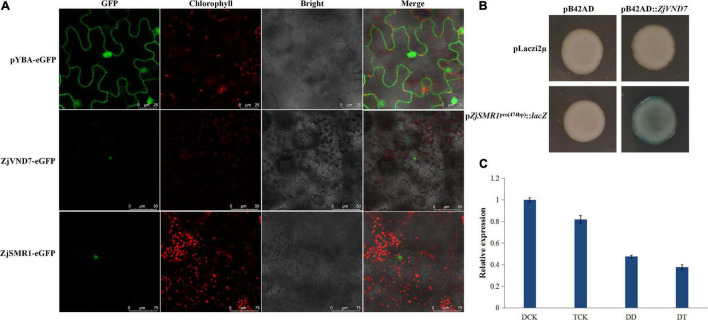
Presumed binding of ZjVND7 to *ZjSMR1* promoter. **(A)** Cellular localization of *ZjSMR1* and *ZjVND7*. **(B)** Yeast one-hybrid validation of the interaction of ZjVND7 and the 474 bp promoter region of *ZjSMR1*. **(C)** Expression level of ZjSMR1 in diploid and autotetraploid plants under drought stress.

To clarify the cause of vessel cell and parenchyma cell proliferation in autotetraploid sour jujube xylem, we investigated whether there were genes that are related to cell division and differentiation and that were also targets of *ZjVND7*. On the basis of the DAP-seq data, *ZjSMR1*, which was annotated to cell division and DNA endoreduplication was recognized as a ZjVND7 downstream target. However, this gene was detected for only one duplication of diploids (with a peak score value of 20, Supplementary Table 1) and for two duplications of autotetraploid with signal values of 53 and 63 (Supplementary Table 1), respectively. In addition, the cellular localization of *ZjSMR1* and *ZjVND7* was determined. Both *ZjVND7* and *ZjSMR1* were expressed in the nucleus, indicating the possibility of space interaction with *ZjVND7* and *ZjSMR1* in Figure 6A. To further verify that ZjVND7 targets *ZjSMR1*, the full-length CDS of *ZjVND7* was fused into a yeast pB42AD vector, and a 474 bp promoter region of *ZjSMR1* was fused into a pLaczi2μ vector to generate a yeast one-hybrid construct. The yeast strains of the three negative controls did not exhibit blue, while the cotransformation of pB42AD:*ZjVND7* and p*ZjSMR1*^pro(474 bp)^:*lacZ* resulted in a blue color (Figure 6B). Moreover, *ZjSMR1* was downregulated under drought stress in both sour jujube ploidy types, which was consistent with the expression trend of *ZjVND7* (Figure 6C). Taken together, these results imply that the ZjVND7 protein can positively target *ZjSMR1*.

Through an analysis of the *cis*-elements in the *ZjSMR1* promoter, the ZjVND7-targeted ACGTG detected in autotetraploids was found to be an ABRE sequence (Supplementary Figure 4). Therefore, we hypothesized that the interaction between *ZjSMR1* and *ZjVND7* might occur as a response to ABA. The expression levels of *ZjSMR1* and *ZjVND7* were measured after ABA was sprayed on diploid sour jujube. The results showed that both *ZjSMR1* and *ZjVND7* were responsive to ABA treatment. The expression levels of both *ZjSMR1* and *ZjVND7* decreased significantly at 2 h after treatment and then increased at 12 h after treatment (Supplementary Figure 5).

## Discussion

Due to frequent modifications to the plant genome after polyploidization, factors affecting polyploid trait variation occur at multiple levels. At the transcriptional or posttranscriptional level, changes in alternative splicing patterns (Zhou et al., 2011), diversification of differentially expressed genes (Wei et al., 2019), and increases in the types and numbers of miRNAs after genome expansion regulate variations in plant adaptability (Liu and Sun, 2017). In addition, an increasing number of studies have confirmed that polyploidization induces changes in epigenetic modifications, including DNA methylation, histone modification, and chromosome remodeling (Ding and Chen, 2018). These epigenetic modifications have been reported in autotetraploid rice (Zhang et al., 2015), switchgrass (Yan et al., 2019), and wheat (Liu et al., 2021). Previous studies have shown that autotetraploid sour jujube at the seedling stage has advantages in terms of phenotype, photosynthesis-related physiology, and adaptability (Li et al., 2019b,c, 2021b). The pathways in response to drought and salt stress in polyploids are more diverse than those in diploids. In the present study, the expression differences of *ZjVND7* between the autotetraploid and diploid plants under drought stress may be the result of responses to alternative splicing, microRNAs, DNA methylation, and histone modifications after polyploidization. Moreover, a gene encoding *S*-adenosylmethionine-dependent methyltransferase expressed in autotetraploid sour jujube suggesting that DNA methylation may occur after polyploidization (Figure 5). These results imply that the mutation caused by whole-genome duplication may be an effective way to maintain genome homeostasis and survival.

In the TF regulation network, TFBSs, which are located in the promoter regions of genes, undergo a series of epigenetic regulatory mechanisms, such as nucleosome localization, histone modification, and DNA methylation, that affect the binding of TFs (Bell et al., 2011; Liu et al., 2013; Kribelbauer et al., 2019). Among them, DNA methylation of the CpG-rich promoter prevents TFs from binding to TFBSs (Bird, 2002), which alters plant traits (Gao et al., 2016). O’Malley et al. (2016) also reported that mC-all and mCG-only methylation impacted binding across all TF families. In particular, E2F family member DEL2 preferentially bound to methylated motifs (Harashima et al., 2013). In addition, changes in H3K3, H3K4, H3K9, and H3K27 modifications in polyploids have been shown cause differences in substance accumulation, photosynthetic capacity, and flowering time (Wang et al., 2006; Ni et al., 2009; Zhang et al., 2020). In the present study, the difference of targets after polyploidization could be explained from the standard DAP-seq libraries used in diploid and autotetraploid sour jujube; these libraries include secondary epigenetic modifications such as cytosine methylation, and these secondary epigenetic modifications. Such subtle targeted differences may be closely related to the growth and metabolic characteristics of diploid and autotetraploid sour jujube.

Many studies have shown that *VND7* has various functions and plays a role in the differentiation of a wide range of xylem vessels, including protoxylem, metaxylem, and fibers, in several different tissues (Yamaguchi et al., 2011). Dong et al. (2020) reported that the mutation in a *VND* homolog *NUT1* causes the thickness of the vessel wall in the protoxylem to decrease and that water transport is blocked during flowering. In the autotetraploid sour jujube, ZjVND7 targeted genes encoding endoglucanase were expressed at a greater level than in the diploids, suggesting that the endoglucanase gene in autotetraploids may be regulated by *ZjVND7*, which may further regulate cell growth and vascular differentiation (Tsabary et al., 2003; Shani et al., 2006). This could further play a greater role in autotetraploids in the response to drought stress. Most studies in plant polyploids have concluded that polyploidization is accompanied by an increase in cell volume, albeit not proportionally in all tissues (Katagiri et al., 2016). In this study, the significant increase in the number of vessels in autotetraploids compared with diploids is speculated to be induced by the greater number of parenchyma cells in xylem, suggesting that autotetraploid sour jujube have a strong capacity for transdifferentiation into xylem vessel elements. Moreover, this process may be regulated by *ZjVND7* (Yamaguchi et al., 2010). Therefore, under the condition of water shortage, autotetraploids have lower hydraulic resistance to water transport than diploids do, which may be one of the reasons for the improved drought tolerance of the autotetraploids.

The greater number of xylem vessels and parenchyma cells in autotetraploid sour jujube is further speculated to be related to the target genes associated with cell division. As a supplement to WEE1, inhibitor of premature vascular differentiation, the members of the plant-specific *SMR* gene family play a suppressive role in cell cycle checkpoint activation (Cools et al., 2011). The rapid induction of *Arabidopsis SMR1* has been proven to occur in response to drought stress, and *SMR1* reduces cell division in the roots and leaves and pushes dividing cells toward expansion, which ultimately leads to an effect on root and leaf growth (Dubois et al., 2018). The hairy body was produced by the *SMR1* overexpression line with an increased amount of nuclear DNA, while the huge endoreplication epidermal cells on the abaxial sides of the sepals were lacking in *smr1*/*lgo* mutants. These data confirm that *SMR1* is an important gene that promotes the transformation of cells division into endoreduplication (Roeder et al., 2010). In the present study, the regulation of putative vessel development-related ZjVND7 to *ZjSMR1* may explain the formation of more xylem parenchyma cells in the veins of autotetraploids than in those of diploids, which is a prerequisite for the greater number of vessels in autotetraploid veins. In other words, the role of *ZjVND7* in xylem vessel differentiation may be partially regulated by *ZjSMR1*. Furthermore, the stronger binding signal in autotetraploids may be due to the difference in *SMR1* motif binding between the autotetraploid and diploid plants, or to changes in the spatial structure resulting from the epigenetic modification of the promoter region. In addition, biotic and abiotic stress signals activate the signaling cascades in plants, triggering the production of reactive oxygen species and the accumulation of antimitotic hormones such as ABA and jasmonic acid. These signaling molecules also stimulate cell cycle checkpoints, resulting in impaired cell cycle G1-to-S transition; DNA replication slows down, and/or delays entry into mitosis (Reichheld et al., 2002; Swiatek et al., 2002; Kadota et al., 2005). *SMRs* respond to abiotic stress by prolonging the cell cycle and regulating endoreplication, as has been reported in *Arabidopsis* (Yi et al., 2014), rice (Peres et al., 2007), and maize (Li et al., 2019a). The binding of ZjVND7 to *ZjSMR1* may be one of the reasons for the changes in *ZjSMR1* expression, and cells may regulate the expression of *ZjSMR1* to prolong the cell cycle and regulate endoreplication, thus improving drought tolerance. In addition, we found that *ZjVND7* and *ZjSMR1* were responsive to ABA with the same expression trend, implying a positive regulatory effect (Supplementary Figure 5). Moreover, the targeted ABRE sequence detected in autotetraploids indicated that the binding of ZjVND7 and *ZjSMR1* may occur in response to the accumulation of antimitotic ABA and could further promote differences in vessel differentiation between diploid and autotetraploid plants (Ramachandran et al., 2021).

It is very difficult to determine whether just the secondary modifications around TFBS cause differences in gene interactions in polyploid. Recently, the DNA 3D structure or “DNA shape” characterized by the physical characteristics of large or small DNA grooves has been considered to affect TF binding (Slattery et al., 2014). However, whether these characteristics change after polyploidization needs to be further researched. Our results also provide information about many target genes functioning in xylem cell differentiation, including unique sour jujube genes without evident homologous genes in the *Arabidopsis* genome. These genes may not be conserved, and their unique significance in sour jujube needs to be further explored.

## Conclusion

**FIGURE 7:**
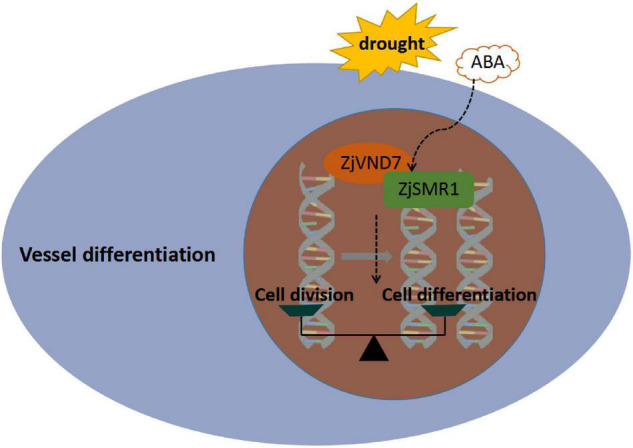
Putative pathway through which ZjVND7 targets *ZjSMR1* in response to drought and ABA.

In present study, a possible functional relationship was proposed (Figure 7). ZjVND7 targeting *ZjSMR1* might regulate transdifferentiation from parenchyma cells into vessels in the xylem. This result might support the balancing effect between cell division and cell differentiation of xylem parenchyma, which can promote vessel differentiation by the division and enlargement of the xylem parenchyma. Therefore, more parenchyma cells were formed in autotetraploids than in diploids, and more vessel cells differentiated to mediate water transport. Moreover, ZjVND7 may regulate the expression of *ZjSMR1* to prolong the cell cycle and regulate endoreplication in response to drought stress and ABA, which may be more convincing after whole-genome duplication. However, these results are hypotheses, and the specific regulatory mechanism needs further verification.

## Data Availability Statement

The original contributions presented in the study are included in the article/Supplementary Material, further inquiries can be directed to the corresponding author/s.

## Author Contributions

YL conceived and designed the experiments, obtained the funding, and is responsible for this article. ML, LH, CZ, and WY conducted the experiments. ML, XL, and HZ collected and analyzed the data. ML wrote the manuscript. XP and YL provided the valuable suggestions on the manuscript. All authors read and approved the final manuscript.

## Conflict of Interest

The authors declare that the research was conducted in the absence of any commercial or financial relationships that could be construed as a potential conflict of interest.

## Publisher’s Note

All claims expressed in this article are solely those of the authors and do not necessarily represent those of their affiliated organizations, or those of the publisher, the editors and the reviewers. Any product that may be evaluated in this article, or claim that may be made by its manufacturer, is not guaranteed or endorsed by the publisher.

## Footnotes

1
https://www.arabidopsis.org/
