# Supplementary material for: Genome-Wide Identification of Direct Targets of ZjVND7 Reveals the Putative Roles of Whole-Genome Duplication in Sour Jujube in Regulating Xylem Vessel Differentiation and Drought Tolerance
Source: Front Plant Sci. 2022 Feb 4;13:829765. doi: 10.3389/fpls.2022.829765 (PMC8854171; doi:10.3389/fpls.2022.829765)
Supplement: Supplementary file 1 [file Data_Sheet_1.docx]

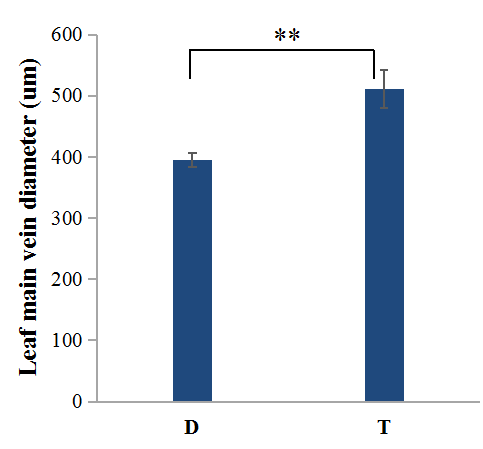


**Supplementary Figure 1** Diameter of leaf main vein from diploid and autotetraploid sour jujube


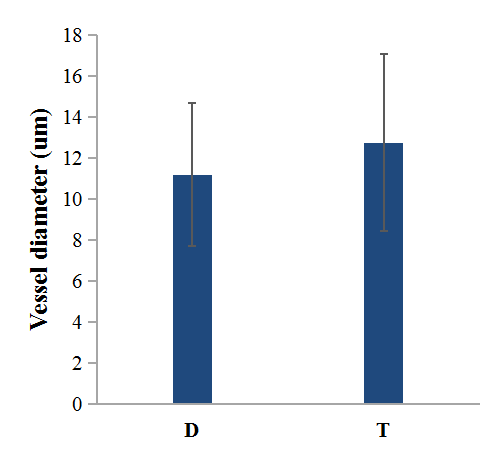


**Supplementary Figure 2** Xylem vessel diameter of diploid and autotetraploid sour jujube veins


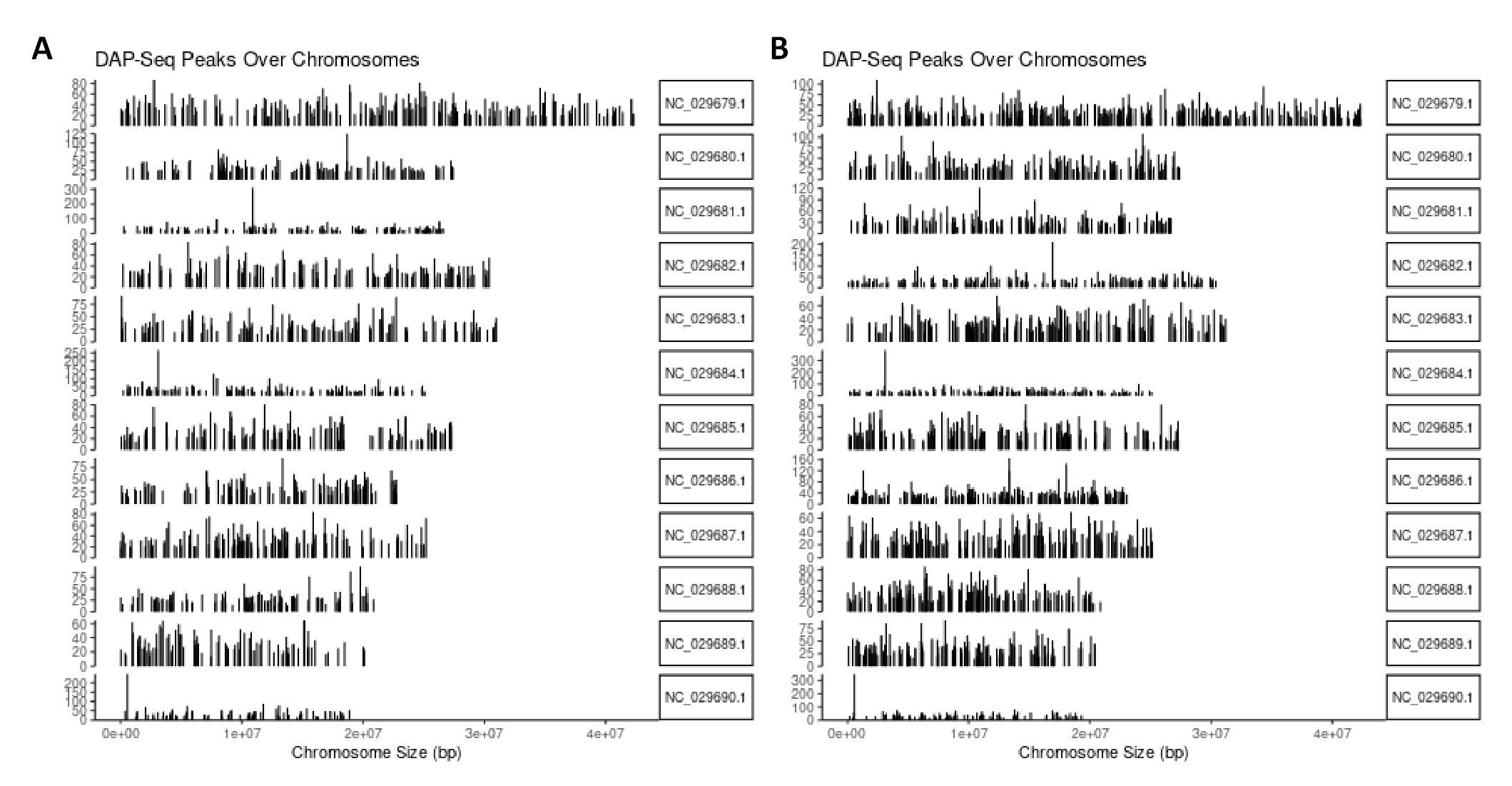


**Supplementary Figure 3** Distribution of peaks on 12 chromosomes of diploid (**A**) and autotetraploid (**B**) sour jujube.


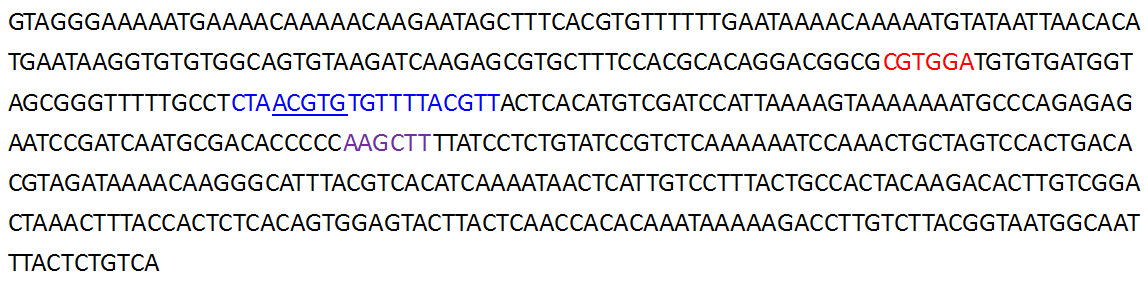


**Supplementary Figure 4** The 474 bp sequence in *SMR1* promoter for yeast one-hybrid assay. The red font is the ZjVND7 binding site detected in diploid, the blue font is the binding site detected in the autotetraploid, and the purple font is the binding site detected for both diploids and autotetraploids.

**
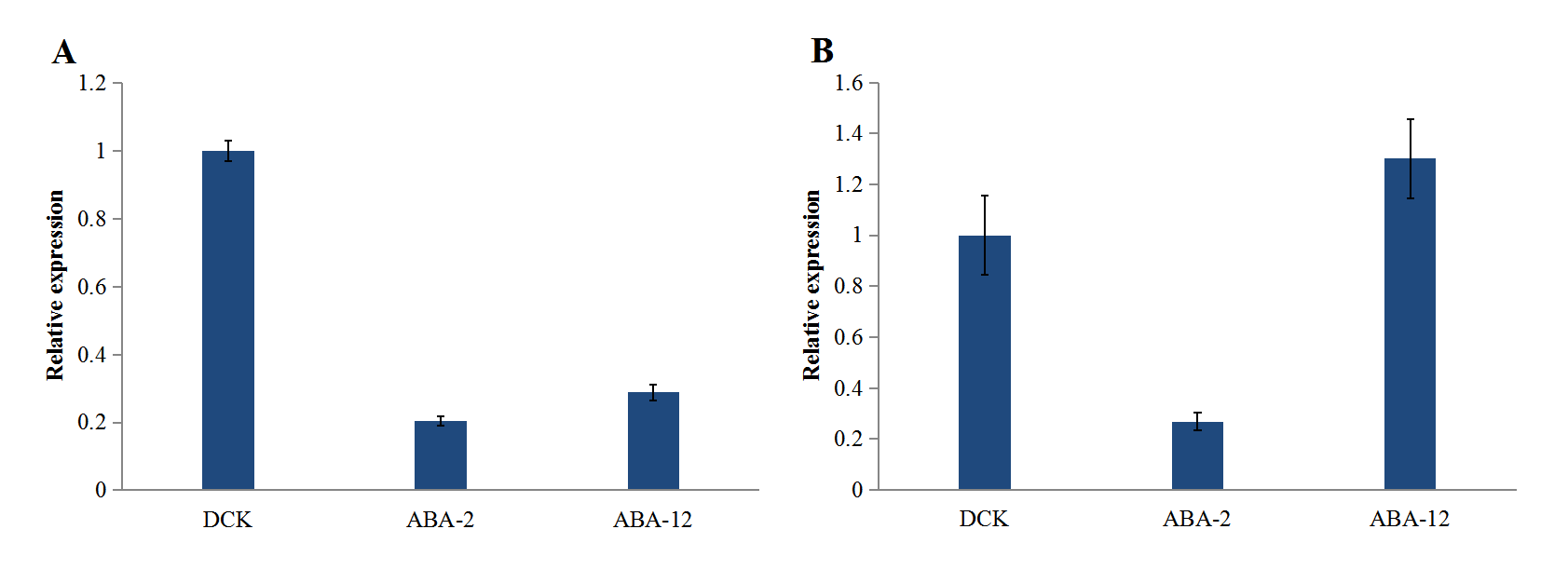
**

**Supplementary Figure 5** Expression level of *ZjVND7* (**A**) and *ZjSMR1* (**B**) in diploid sour jujube after ABA treatment.
