# Supplementary material for: Genome-Wide Identification of Direct Targets of ZjVND7 Reveals the Putative Roles of Whole-Genome Duplication in Sour Jujube in Regulating Xylem Vessel Differentiation and Drought Tolerance
Source: Front Plant Sci. 2022 Feb 4;13:829765. doi: 10.3389/fpls.2022.829765 (PMC8854171; doi:10.3389/fpls.2022.829765)
Supplement: Supplementary file 4 [file Table_3.docx]

Supplementary Table 3 Five types of motif in diploid and autotetraploid

| Motif  type | Diploid | Autotetraploid | E-value | |
| --- | --- | --- | --- | --- |
|  |  |  | Diploid | Autotetraploid |
| Motif 1 | 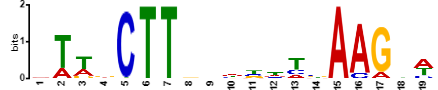 | 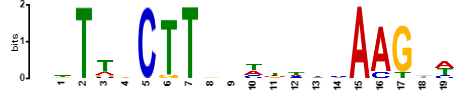 | 1.3e-083 | 7.5e-156 |
|  | 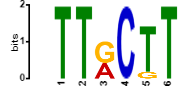 | 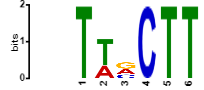 |  |  |
|  | 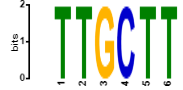 | 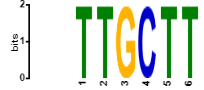 |  |  |
|  |  | 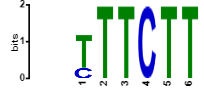 |  |  |
|  |  | 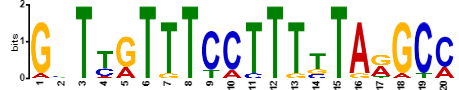 |  |  |
| Motif 2 | 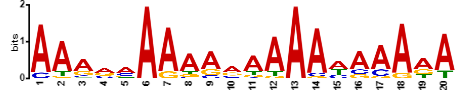 | 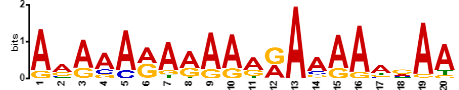 | 1.4e-043 | 6.1e-025 |
| Motif 3 | 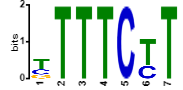 | 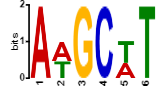 | 1.3e-009 | 4.4e-007 |
| Motif 4 | 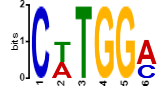 | 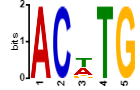 | 1.9e-006 | 3.4e-005 |
| Motif 5 | 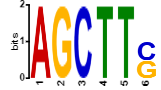 | 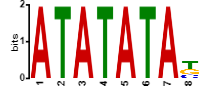 | 1.7e-002 | 2.0e-004 |
